# Encephalopathy following subcutaneous administration of Brixadi® extended-release buprenorphine

**DOI:** 10.1016/j.toxrep.2026.102320

**Published:** 2026-07-22

**Authors:** Justin Seltzer, Riku Moriguchi, Kara Yeung, Yuri Shindo, Jeremy Hardin, Henrik Galust, Alicia Minns, Richard Clark

**Affiliations:** a Division of Medical Toxicology, Department of Emergency Medicine, University of California San Diego, San Diego, CA, USA; b Kaiser Permanente Medical Group, San Diego, CA, USA; c Department of Emergency Medicine, Stanford University, Palo Alto, CA, USA; d Division of Hospital Medicine, Department of Medicine, University of California San Diego, San Diego, CA, USA; e Department of Emergency Medicine, Eisenhower Medical Center, Rancho Mirage, CA, USA

**Keywords:** Buprenorphine, Substance use disorder, Overdose, Long-acting injectable, Opioid

## Abstract

Long-acting injectable buprenorphine is one option for the management of opioid use disorder. The United States Food and Drug Administration has approved two brands for monthly injection, Sublocade ® (Indivior, North Chesterfield, VA, USA) and Brixadi® (Braeburn, Plymouth Meeting, PA, USA). We report the development of encephalopathy following administration of Brixadi®. Consent was obtained from the patient. A 62-year-old patient with a history of current methamphetamine and prior fentanyl abuse, chronic pain due to lumbar radiculopathy presented to the emergency department with three days of altered mental status. She received subcutaneous Brixadi® 128 mg monthly formulation three days prior at an outpatient addiction medicine clinic, reportedly to treat simultaneously presumptive incidental opioid dependence from contaminated methamphetamine use due to serial positive urine fentanyl testing over the prior three visits, most recently 3 months prior, and chronic low back pain. Urine fentanyl testing on the date of presentation was negative. She developed persistent altered mentation and somnolence starting two hours after injection.

## Highlights

• Toxicity from injectable, long-acting buprenorphine is rare.
• Presentation may differ from other opioids, likely due to partial agonist effects.
• A prolonged recovery time is expected due to the long-acting formulation..

## Graphical Abstract

**Figure:**
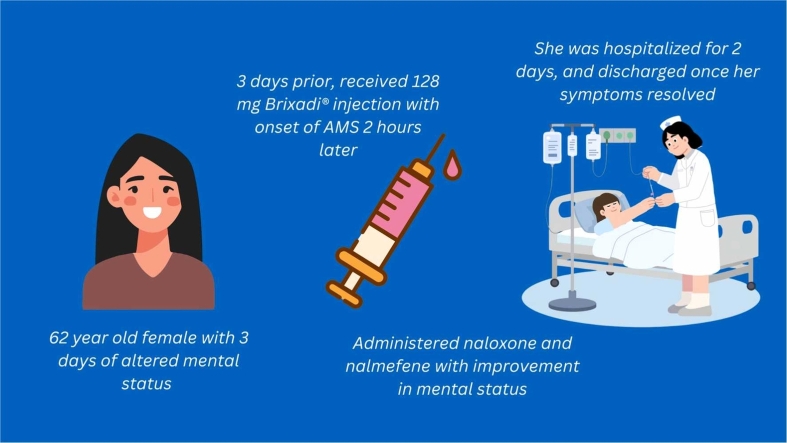

On initial evaluation in the emergency department, vital signs were unremarkable and her examination was notable for somnolence and mild disorientation. Blood testing and neuroimaging were unremarkable. Urine drug testing was positive for amphetamines and negative for barbiturates, cocaine metabolites, opiates, methadone, fentanyl, oxycodone, phencyclidine, and tetrahydrocannabinol metabolites. A serum free buprenorphine concentration, obtained 18 h after arrival, was 2.4 ng/mL.

The patient was admitted and the next morning developed bradypnea, miosis, and worsened encephalopathy. Due to concern for buprenorphine-induced encephalopathy, naloxone and nalmefene were trialed with temporary improvement. The next day, the patient’s mental status normalized and she was discharged. At four months follow up, she had no residual effects noted.

This case highlights that buprenorphine toxicity should be considered in circumstances of encephalopathy of unclear etiology following long-acting buprenorphine injection, especially once other major potential causes have been ruled out.

## 1 Introduction

Long-acting injectable buprenorphine is a well-established method of managing opioid use disorder. Use of injectable buprenorphine, as opposed to sublingual or oral preparations, is thought to improve long term opioid use disorder treatment compliance [1], [2]. In the United States, two long-acting injectable forms are available for monthly subcutaneous injection, Sublocade ® (Indivior, North Chesterfield, VA, USA) and Brixadi® (Braeburn, Plymouth Meeting, PA, USA); Brixadi® also has a weekly formulation. Though buprenorphine can be used for management of both opioid use disorder and chronic pain, these long-acting formulations are not Food and Drug Administration approved in the United States for chronic pain management [2], [3].

We report the case of a patient who developed encephalopathy following outpatient administration of the monthly formulation of Brixadi®, Written consent to publish the case details was obtained from the patient.

## 2 Case report

A 62-year-old female with a history of current methamphetamine and prior fentanyl abuse presented to the emergency department with three days of altered mental status following administration of Brixadi® monthly formulation three days prior. She initially presented to an outpatient addiction medicine clinic for a routine visit for methamphetamine use disorder. The clinic physician noted positive in-office fentanyl testing for the past three visits, the most recent of which was three months prior, which was felt to be due to unintentional fentanyl exposure from contaminated methamphetamine. Though the patient had a prior history of opioid use and overdose, she denied current opioid use. Given this and the patient’s complaint of uncontrolled chronic back pain, the clinic physician administered one dose of buprenorphine-naloxone 4–1 mg sublingually and, following an unremarkable two hour observation period, she received 128 mg of Brixadi® monthly formulation subcutaneously.

The clinic physician contacted the patient’s partner for routine follow up four days later, who noted she “has been walking slowly and been incoherent” since approximately two hours after the Brixadi® injection. Given concern for toxicity, the clinic physician recommended emergency department evaluation at that time.

On initial emergency department evaluation, the patient was noted to be somnolent but responsive to stimulation, with orientation to name and location only. Her pupils were mid-range, equal, and reactive. She had an unremarkable cardiopulmonary examination. Initial vital signs were blood pressure 144/85 mmHg, respiratory rate 20 breaths per minute, heart rate 106 beats per minute, oxygen saturation of 93% on room air, and temperature 97.8 °F.

Laboratory testing with complete blood count, comprehensive metabolic panel, thyroid stimulating hormone, ammonia, and venous blood gas revealed no relevant abnormalities to explain her encephalopathy. Salicylate, acetaminophen, and serum ethanol testing were negative. Computed tomography of the head revealed no acute findings. Lumbar puncture was attempted in the emergency department but was not successful; no further attempts were made during her inpatient stay without documented rationale. Urinalysis was unremarkable. Urine drugs of abuse screening was positive for amphetamines and confirmed to be methamphetamine by liquid chromatography with tandem mass spectrometry. Otherwise, urine testing was negative for barbiturates, cocaine metabolites, opiates, methadone, fentanyl, oxycodone, phencyclidine, and tetrahydrocannabinol metabolites. Of note, these results were unchanged from urine drug screening results obtained seven days prior to presentation. A serum free buprenorphine concentration from 18 h after arrival, was 2.4 ng/mL; the expected therapeutic steady state concentration of monthly Brixadi® is 2–3.9 ng/mL [4].

She received trial doses of 0.1 mg and 1 mg intravenous naloxone in the emergency department without clinical improvement and was admitted to the hospital.

The following morning, she developed mild bradypnea (10 breaths/minute), worsening encephalopathy, and pupillary miosis, with improvement following intravenous naloxone 0.5 mg. However, this effect was not sustained. Medical toxicology was consulted and recommended a trial of intravenous nalmefene 0.5 mg in lieu of naloxone infusion due to the stronger opioid receptor binding affinity of nalmefene compared to naloxone. Improvement in her mental status and respiratory rate were noted, with sustained effect over approximately three hours. No additional opioid antagonists were subsequently administered.

**Fig. 1:**
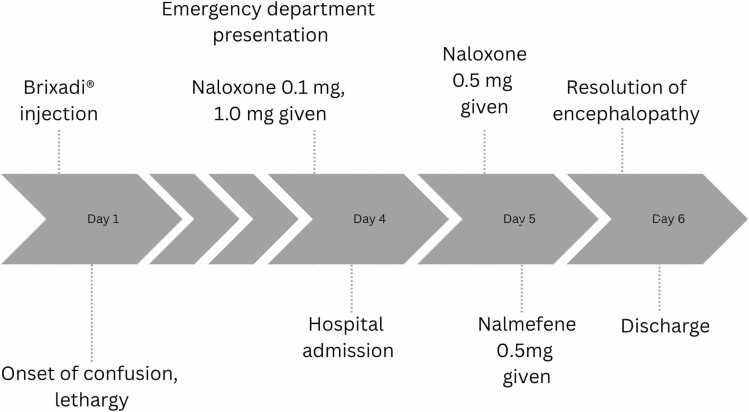
Timeline of clinical events

The following day, the patient awoke with normalized mental status and was discharged home the same day. No further inpatient testing was performed. The timeline of events can be viewed in Fig. 1.

Outpatient brain MRI obtained 20 days after discharge showed no acute abnormalities. At her next primary care follow up visit four months after discharge, the patient had no noted residual effects.

## 3 Discussion

This case highlights the potential adverse effects of long-acting buprenorphine injection. This case is, to our knowledge, the second reported case of buprenorphine toxicity from a long-acting formulation to be reported in the literature [5].

We suspect the patient’s recent lack of opioid exposure and likely associated loss of tolerance account for the development of toxicity. Though she had a past history of opioid use, she last had urine evidence of opioid exposure three months prior to injection; drug screening was negative for opioids the day of injection as well as on presentation.

The patient’s initial presentation was inconsistent with a classic opioid toxidrome, lacking the typical miosis or respiratory depression at the outset. Prior reports of accidental buprenorphine exposure have also noted some atypical findings not classically associated with opioids [6]. Interestingly, these findings developed the morning after presentation, days after the initial exposure.

We are unable to explain these findings on mechanistic or pharmacologic grounds. The serum buprenorphine concentration obtained at the time of her initial evaluation was within range of the manufacturer’s expected steady state concentration [4]. While the well-established “ceiling effect” of buprenorphine due to partial µ opioid receptor agonism may limit some clinical findings of opioid toxicity, this does not explain her initial presentation nor the delayed presentation of her classic opioid toxidrome [7], [8].

It is unclear why her mental status rapidly normalized, as we would expect a slow return to baseline as was noted in the published cases [5], [6]. The buprenorphine injection itself was well documented and the patient had no known coincidental exposures, with onset of symptoms a short time after injection.

An alternative cause of her presentation that was not identified during her hospital stay is possible. The lack of cerebrospinal fluid analysis, brain MRI, or electroencephalogram to rule out major potential confounders such as seizures or central nervous system infection or inflammatory condition are major limitations to this report. However, the temporal relationship between her symptoms and Brixadi® injection as well as response of her encephalopathy to opioid antagonists support buprenorphine toxicity as the most likely cause of her presentation.

In summary, we present a case of encephalopathy and delayed opioid toxicity occurring after administration long-acting injectable buprenorphine. Her presentation was atypical compared to established presentations of opioid overdose, though the literature on how long-acting injectable buprenorphine toxicity presents is sparse. Fortunately, the patient’s symptoms responded to opioid antagonists and resolved without further interventions.

Providers should consider buprenorphine toxicity along with other potential causes of encephalopathy for patients presenting with altered mentation of unclear etiology in the setting of long-acting buprenorphine injection. Toxicity from long-acting buprenorphine may have an atypical presentation compared to toxicity from other opioids.

## CRediT authorship contribution statement

**Alicia Minns:** Writing – review & editing. **Jeremy Hardin:** Writing – review & editing. **Richard Clark:** Writing – review & editing, Supervision. **Riku Moriguchi:** Writing – review & editing, Writing – original draft, Project administration, Formal analysis, Data curation, Conceptualization. **Justin Seltzer:** Writing – review & editing, Writing – original draft, Project administration, Investigation, Formal analysis, Data curation, Conceptualization. **Yuri Shindo:** Writing – review & editing. **Kara Yeung:** Writing – review & editing.

## Declaration of Competing Interest

The authors declare the following financial interests/personal relationships which may be considered as potential competing interests: Dr. Seltzer received a research grant with associated salary support from Purdue Pharma L.P., manufacturer of nalmefene HCl injection, to study nalmefene in the emergency department setting.

## Data Availability

The data that has been used is confidential.
